# Supplementary material for: Enhancement of antiphotoaging properties of Cannabis sativa stem water extracts by fermentation with Lacticaseibacillus casei
Source: PLoS One. 2025 Aug 14;20(8):e0329634. doi: 10.1371/journal.pone.0329634 (PMC12352839; doi:10.1371/journal.pone.0329634)
Supplement: S1 Data — This dataset includes raw absorbance-derived cell viability percentages of human dermal fibroblasts (HDFs) treated with various concentrations (0, 100, 250, 500, and 1000 μg/mL) of Cannabis sativa stem extracts, non-fermented (0 h) and fermented (48 h, 72 h, 96 h), as well as Lacticaseibacillus casei culture supernatant (LC Sup), Gifu Anaerobic Medium (GAM), and ascorbic acid. Viability was assessed by MTT assay after 24 h treatment without UVB exposure. Data are presented as individual replicate values (n = 3) and corresponding mean ± standard deviation for each condition. (PDF) [file pone.0329634.s001.pdf]

Supporting information

S1 Data. Raw data for Fig 1 (MTT-based cell viability assay).

Sample	Concentration (µg/mL)	Replicate 1 (%)	Replicate 2 (%)	Replicate 3 (%)	Mean ± SD (%)
Ascorbic acid	0.0	1.20	1.26	1.32	1.25 ±0.06
	3.9	1.22	1.34	1.28	1.28 ±0.06
	7.8	1.22	1.31	1.28	1.27 ±0.05
	15.6	1.32	1.28	1.29	1.30 ±0.02
	31.3	1.28	1.30	1.27	1.28 ±0.01
	62.5	1.29	1.28	1.25	1.27 ±0.02
	125.0	1.27	1.24	1.26	1.25 ±0.01
	250.0	1.24	1.23	1.25	1.24 ±0.01
	500.0	0.61	0.64	0.63	0.63 ±0.02
	1,000.0	0.39	0.34	0.34	0.36 ±0.03
GAM	0.0	1.26	1.27	1.29	1.28 ± 0.01
	3.9	1.30	1.26	1.25	1.27 ± 0.02
	7.8	1.28	1.25	1.29	1.27 ± 0.02
	15.6	1.31	1.26	1.27	1.28 ± 0.03
	31.3	1.31	1.28	1.32	1.30 ± 0.02
	62.5	1.34	1.37	1.33	1.35 ± 0.02
	125.0	1.40	1.39	1.39	1.39 ± 0.01
	250.0	1.45	1.45	1.45	1.45 ± 0.00
	500.0	1.53	1.50	1.50	1.51 ± 0.02
	1,000.0	1.58	1.61	1.61	1.60 ±0.02
0 h	0.0	1.42	1.47	1.46	1.45 ±0.03
	3.9	1.25	1.49	1.40	1.38 ±0.12
	7.8	1.25	1.36	1.33	1.31 ±0.06
	15.6	1.29	1.30	1.28	1.29 ±0.01
	31.3	1.29	1.30	1.27	1.29 ±0.01
	62.5	1.38	1.39	1.34	1.37 ±0.03
	125.0	1.40	1.40	1.42	1.40 ±0.01
	250.0	1.39	1.42	1.40	1.40 ±0.02
	500.0	1.40	1.39	1.41	1.40 ±0.01
	1,000.0	1.43	1.46	1.45	1.45 ±0.01
48 h	0.0	1.42	1.35	1.42	1.40 ±0.05
	3.9	1.48	1.41	1.39	1.43 ±0.05
	7.8	1.31	1.37	1.30	1.33 ±0.04
	15.6	1.34	1.35	1.30	1.33 ±0.02
	31.3	1.23	1.30	1.27	1.27 ±0.04
	62.5	1.17	1.31	1.25	1.25 ±0.07
	125.0	1.23	1.24	1.22	1.23 ±0.01
	250.0	1.30	1.31	1.36	1.32 ±0.03
	500.0	1.34	1.35	1.32	1.34 ±0.01
	1,000.0	1.37	1.40	1.39	1.39 ±0.02
72 h	0.0	1.30	1.36	1.36	1.34 ±0.03
	3.9	1.21	1.30	1.35	1.28 ±0.07
	7.8	1.19	1.24	1.25	1.23 ±0.03
	15.6	1.19	1.19	1.20	1.19 ±0.00
	31.3	1.18	1.18	1.20	1.19 ±0.01
	62.5	1.18	1.19	1.19	1.19 ±0.01

	125.0	1.19	1.22	1.22	1.21 ±0.01
	250.0	1.21	1.21	1.20	1.21 ±0.01
	500.0	1.26	1.26	1.27	1.27 ±0.01
	1,000.0	1.30	1.28	1.29	1.29 ±0.01
96 h	0.0	1.39	1.28	1.24	1.30 ± 0.08
	3.9	1.29	1.22	1.25	1.26 ± 0.04
	7.8	1.26	1.23	1.19	1.23 ± 0.04
	15.6	1.21	1.18	1.18	1.19 ± 0.02
	31.3	1.19	1.19	1.16	1.18 ± 0.01
	62.5	1.20	1.17	1.17	1.18 ± 0.02
	125.0	1.19	1.18	1.17	1.18 ± 0.01
	250.0	1.18	1.17	1.21	1.19 ± 0.02
	500.0	1.20	1.22	1.23	1.22 ± 0.01
	1,000.0	1.22	1.24	1.24	1.23 ± 0.01
LC sup	0.0	1.28	1.29	1.28	1.28 ± 0.01
	3.9	1.26	1.27	1.28	1.27 ± 0.01
	7.8	1.24	1.26	1.27	1.26 ± 0.02
	15.6	1.26	1.27	1.28	1.27 ± 0.01
	31.3	1.28	1.28	1.31	1.29 ± 0.02
	62.5	1.30	1.32	1.33	1.32 ± 0.02
	125.0	1.36	1.39	1.39	1.38 ± 0.02
	250.0	1.45	1.47	1.48	1.47 ± 0.01
	500.0	1.53	1.51	1.55	1.53 ± 0.02
	1,000.0	1.60	1.63	1.64	1.62 ± 0.02

This dataset includes raw absorbance-derived cell viability percentages of human dermal fibroblasts (HDFs) treated with various concentrations (0, 100, 250, 500, and 1000 μg/mL) of *Cannabis sativa* stem extracts, non-fermented (0 h) and fermented (48 h, 72 h, 96 h), as well as *Lacticaseibacillus casei* culture supernatant (LC Sup), Gifu Anaerobic Medium (GAM), and ascorbic acid. Viability was assessed by MTT assay after 24 h treatment without UVB exposure. Data are presented as individual replicate values (n = 3) and corresponding mean ± standard deviation for each condition.
