# Supplementary material for: Enhancement of antiphotoaging properties of Cannabis sativa stem water extracts by fermentation with Lacticaseibacillus casei
Source: PLoS One. 2025 Aug 14;20(8):e0329634. doi: 10.1371/journal.pone.0329634 (PMC12352839; doi:10.1371/journal.pone.0329634)
Supplement: S2 Data — This dataset presents the raw absorbance values of HDFs exposed to increasing doses of UVB light (0–70 mJ/cm²) for 24 hours. (PDF) [file pone.0329634.s002.pdf]

Supporting information

S2 Data. Raw data for Supplementary Fig 1 (MTT assay: UVB cytotoxicity).

Replicate	0 mJ/cm^2	25 mJ/cm^2	30 mJ/cm^2	40 mJ/cm^2	50 mJ/cm^2	70 mJ/cm^2
1	0.547	0.5184	0.4823	0.4368	0.4284	0.3824
2	0.5617	0.5288	0.4987	0.4304	0.4389	0.3619
3	0.5585	0.4873	0.4686	0.4529	0.4304	0.3707
4	0.5624	0.524	0.5009	0.4539	0.4455	0.4022
5	0.5636	0.5016	0.4699	0.4633	0.4514	0.4041
6	0.5694	0.5346	0.5165	0.4756	0.4698	0.4232
7	0.5465	0.5211	0.4955	0.4483	0.4402	0.3909
8	0.5564	0.5026	0.4757	0.4439	0.4244	0.3954
9	0.5421	0.5048	0.4858	0.4509	0.4473	0.3939
10	0.5747	0.5051	0.4985	0.4509	0.4502	0.4016
11	0.5783	0.5115	0.4954	0.4486	0.4504	0.4002
12	0.5784	0.5101	0.4975	0.4558	0.4573	0.4183

This dataset presents the raw absorbance values of HDFs exposed to increasing doses of UVB light (0–70 mJ/cm^2) for 24 hours.
