# Supplementary material for: Enhancement of antiphotoaging properties of Cannabis sativa stem water extracts by fermentation with Lacticaseibacillus casei
Source: PLoS One. 2025 Aug 14;20(8):e0329634. doi: 10.1371/journal.pone.0329634 (PMC12352839; doi:10.1371/journal.pone.0329634)
Supplement: S3 Data — This dataset provides the calculated MMP-1 concentrations (ng/mL) in HDFs treated with non-fermented (0 h) and fermented C. sativa stem extracts (48 h, 72 h, 96 h), LC Sup, GAM, and ascorbic acid (positive control), following UVB irradiation at 25 mJ/cm². All treatment groups except the untreated control were UVB-irradiated. MMP-1 levels in culture supernatants were measured 24 hours after treatment. Each condition was tested in triplicate (n = 3), and data are reported as mean ± standard deviation in ng/mL. (PDF) [file pone.0329634.s003.pdf]

Supporting information

S3 Data. Raw data for Fig 3B (MMP-1 ELISA assay).

Group	Replicate 1 (ng/mL)	Replicate 2 (ng/mL)	Replicate 3 (ng/mL)	Mean ± SD (ng/mL)
Con	3112.5	2658.8	3168.0	2979.8 ± 279.4
UVB	5660.3	5557.5	5584.5	5600.8 ± 53.3
AS	3635.3	3743.3	3901.5	3760.0 ± 133.9
GAM	5947.5	5838.0	5522.3	5769.3 ± 220.8
0 h	4663.5	4493.3	4194.8	4450.5 ± 237.3
48 h	5174.3	4638.0	4661.3	4824.5 ± 303.1
72 h	4182.8	4475.3	4943.3	4533.8 ± 383.6
96 h	4929.8	4476.0	4492.5	4632.8 ± 257.3
LC Sup	4409.3	4530.0	4641.8	4527.0 ± 116.3

This dataset provides the calculated MMP-1 concentrations (ng/mL) in HDFs treated with non-fermented (0 h) and fermented *C. sativa* stem extracts (48 h, 72 h, 96 h), LC Sup, GAM, and ascorbic acid (positive control), following UVB irradiation at 25 mJ/cm². All treatment groups except the untreated control were UVB-irradiated. MMP-1 levels in culture supernatants were measured 24 hours after treatment. Each condition was tested in triplicate (n = 3), and data are reported as mean ± standard deviation in ng/mL.
