# Supplementary material for: Enhancement of antiphotoaging properties of Cannabis sativa stem water extracts by fermentation with Lacticaseibacillus casei
Source: PLoS One. 2025 Aug 14;20(8):e0329634. doi: 10.1371/journal.pone.0329634 (PMC12352839; doi:10.1371/journal.pone.0329634)
Supplement: S4 Data — This dataset contains the quantified concentrations (µg/g extract) of quercetin and kaempferol in C. sativa stem extracts fermented for 0 h, 48 h, 72 h, and 96 h. GAM medium and LC Sup were also analyzed. Quantification was performed by HPLC. (PDF) [file pone.0329634.s004.pdf]

Supporting information

S4 Data. Raw data for Supplementary Fig 3 (Quantification of quercetin and kaempferol).

Group		Replicate 1 (µg/g extract)	Replicate 2 (µg/g extract)	Replicate 3 (µg/g extract)	Mean ± SD (µg/g extract)
Quercetin	GAM	0	0	0	0.0 ± 0.0
	0 h	3.1	3.2	3.3	3.2 ± 0.1
	48 h	7.6	7.3	7.9	7.6 ± 0.3
	72 h	11.7	11.0	11.2	11.3 ± 0.6
	96 h	10.3	10.7	10.5	10.5 ± 0.4
	LC Sup	0	0	0	0 ± 0.0
Kampferol	GAM	0	0	0	0 ± 0.0
	0 h	2.2	2.0	2.1	2.1 ± 0.1
	48 h	5.9	5.8	6.0	5.9 ± 0.2
	72 h	8.7	9.0	9.3	9.0 ± 0.3
	96 h	8.2	8.5	8.6	8.4 ± 0.4
	LC Sup	0	0	0	0.0 ± 0.0

This dataset contains the quantified concentrations (µg/g extract) of quercetin and kaempferol in *C. sativa* stem extracts fermented for 0 h, 48 h, 72 h, and 96 h. GAM medium and LC Sup were also analyzed. Quantification was performed by HPLC.
