# Supplementary material for: Enhancement of antiphotoaging properties of Cannabis sativa stem water extracts by fermentation with Lacticaseibacillus casei
Source: PLoS One. 2025 Aug 14;20(8):e0329634. doi: 10.1371/journal.pone.0329634 (PMC12352839; doi:10.1371/journal.pone.0329634)
Supplement: S1 Table — 1, Phospho-SAPK/JNK (Thr183/Tyr185) (G9) Mouse mAb; 2, SAPK/JNK Antibody; 3, Phospho-p44/42 MAPK (Erk1/2) (Thr202/Tyr204) (D13.14.4E) XP® Rabbit mAb; 4, p44/42 MAPK (Erk1/2) (137F5) Rabbit mAb; 5, Phospho-NF-κB p65 (Ser536) (93H1) Rabbit mAb; 6, NF-κB p65 (C22B4) Rabbit mAb; 7, GAPDH (14C10) Rabbit mAb; 8, mouse anti-rabbit IgG-HRP; 9, m-IgGκ BP-HRP. (PDF) [file pone.0329634.s005.pdf]

Supporting information

S1 Table. Antibodies used for western blot analysis

Target	Type	Dilution rate	Brand	Cat. No.
**p-JNK**[1]	Mouse IgG (primary)	1:1000	Cell Signaling	#9255
**JNK**[2]	Rabbit IgG (primary)	1:1000	Cell Signaling	#9252
**p-ERK**[3]	Rabbit IgG (primary)	1:1000	Cell Signaling	#4370
**ERK**[4]	Rabbit IgG (primary)	1:1000	Cell signaling	#4695
**p-p65**[5]	Rabbit IgG (primary)	1:1000	Cell Signaling	#3033
**P65**[6]	Rabbit IgG (primary)	1:1000	Cell Signaling	#4764
**GAPDH**[7]	Rabbit IgG (primary)	1:10000	Cell Signaling	#2118
**Rabbit IgG**[8]	Mouse IgG-HRP (secondary)	1:10000	Santa Cruz Biotechnology	SC-2357
**Mouse IgG**[9]	Mouse IgGκ BP-HRP (secondary)	1:10000	Santa Cruz Biotechnology	SC-516102

1, Phospho-SAPK/JNK (Thr183/Tyr185) (G9) Mouse mAb; 2, SAPK/JNK Antibody; 3, Phospho-p44/42 MAPK (Erk1/2) (Thr202/Tyr204) (D13.14.4E) XP® Rabbit mAb; 4, p44/42 MAPK (Erk1/2) (137F5) Rabbit mAb; 5, Phospho-NF-κB p65 (Ser536) (93H1) Rabbit mAb; 6, NF-κB p65 (C22B4) Rabbit mAb; 7, GAPDH (14C10) Rabbit mAb; 8, mouse anti-rabbit IgG-HRP; 9, m-IgGκ BP-HRP.
