# Supplementary material for: Enhancement of antiphotoaging properties of Cannabis sativa stem water extracts by fermentation with Lacticaseibacillus casei
Source: PLoS One. 2025 Aug 14;20(8):e0329634. doi: 10.1371/journal.pone.0329634 (PMC12352839; doi:10.1371/journal.pone.0329634)
Supplement: S1 Fig — The viability of human dermal fibroblasts was evaluated using an MTT assay. Cells were irradiated with UVB (0–70 mJ/cm2) for 24 h. Data are expressed as the mean ± standard deviation (n = 3 per group). (PDF) [file pone.0329634.s006.pdf]

Supporting information

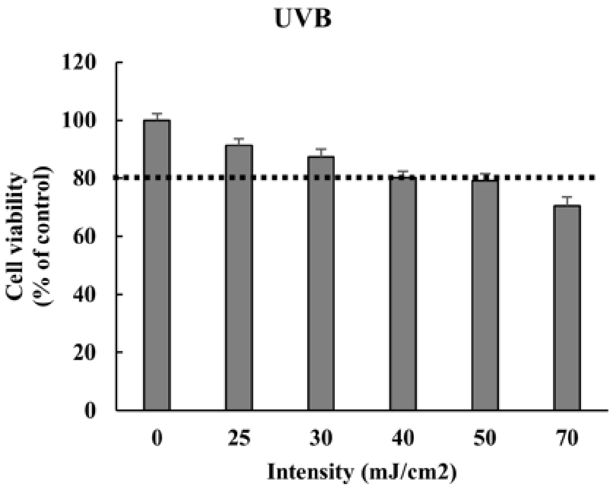

Supplementary Figure 1. Assessment of cytotoxicity of the exposure to UVB light.

The viability of human dermal fibroblasts was evaluated using an MTT assay. Cells were irradiated with UVB (0–70 mJ/cm^2) for 24 h. Data are expressed as the mean ± standard deviation (n = 3 per group).
