# Supplementary material for: Enhancement of antiphotoaging properties of Cannabis sativa stem water extracts by fermentation with Lacticaseibacillus casei
Source: PLoS One. 2025 Aug 14;20(8):e0329634. doi: 10.1371/journal.pone.0329634 (PMC12352839; doi:10.1371/journal.pone.0329634)
Supplement: S2 Fig — HDFs were exposed to UVB (25 mJ/cm²) and treated with C. sativa stem water extracts (0 h, 48 h, 72 h, 96 h), LC Sup, or GAM for 24 h. Protein expression levels were assessed using antibodies against phosphorylated and total forms of ERK, JNK, and p65. GAPDH was used as a loading control. Representative blot images are shown for (A) p-ERK, (B) total ERK, (C) p-JNK, (D) total JNK, (E) p-p65, (F) p65, and (G) GAPDH. Molecular weights are indicated in kilodaltons (kDa). (PDF) [file pone.0329634.s007.pdf]

Supporting information

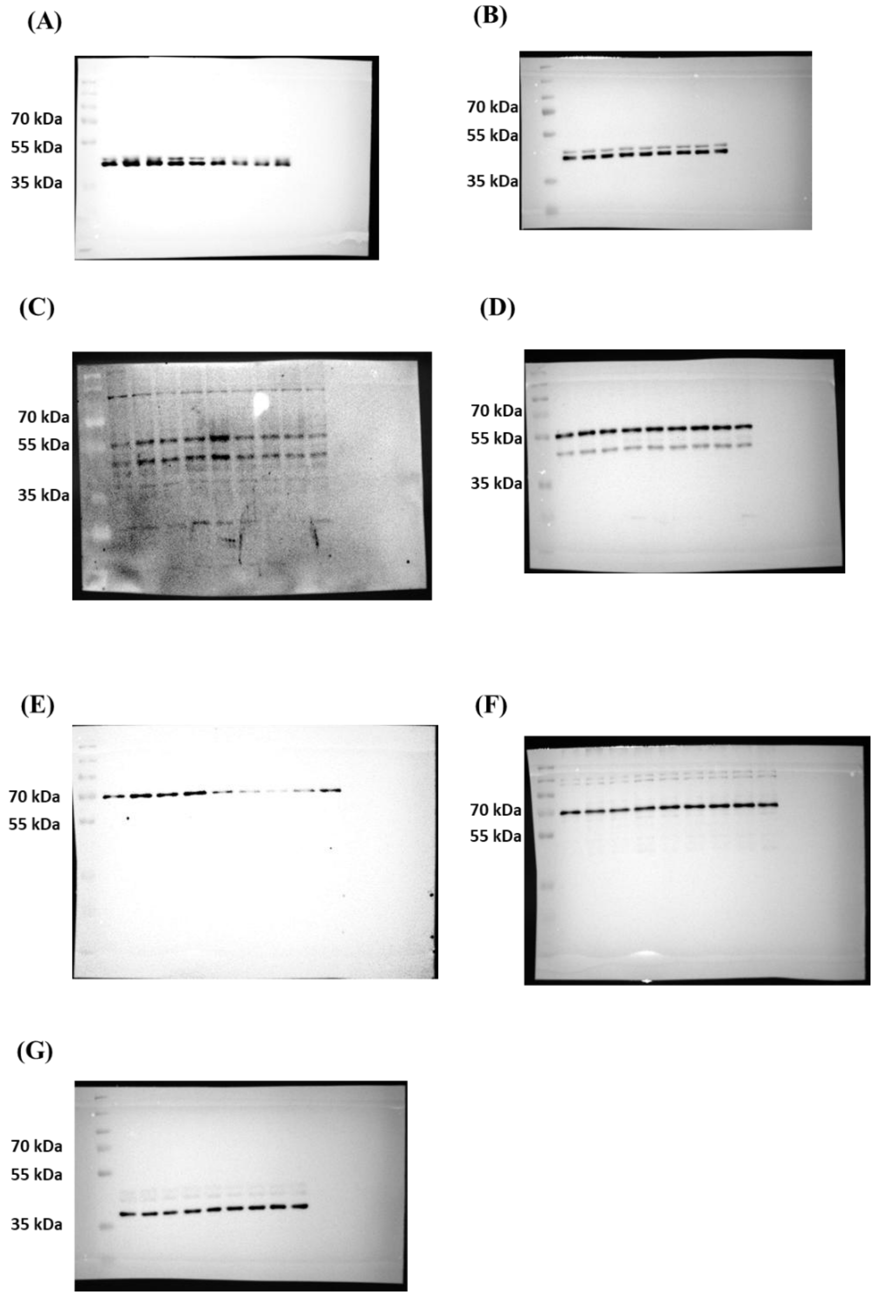

Supplementary Figure 2. Western blot analysis of signaling pathways involved in UVB-induced photoaging. HDFs were exposed to UVB (25 mJ/cm²) and treated with *C. sativa* stem water extracts (0 h, 48 h, 72 h, 96 h), LC Sup, or GAM for 24 h. Protein expression levels were assessed using antibodies against phosphorylated and total forms of ERK, JNK, and p65. GAPDH was used as a loading control. Representative blot images are shown for (A) p-ERK, (B) total ERK, (C) p-JNK, (D) total JNK, (E) p-p65, (F) p65, and (G) GAPDH. Molecular weights are indicated in kilodaltons (kDa).
