# Supplementary material for: Enhancement of antiphotoaging properties of Cannabis sativa stem water extracts by fermentation with Lacticaseibacillus casei
Source: PLoS One. 2025 Aug 14;20(8):e0329634. doi: 10.1371/journal.pone.0329634 (PMC12352839; doi:10.1371/journal.pone.0329634)
Supplement: S3 Fig — The concentrations of quercetin and kaempferol were determined in C. sativa stem extracts fermented for 0 h, 48 h, 72 h, and 96 h, as well as in controls including GAM, LC Sup, and the non-fermented 0 h sample. (PDF) [file pone.0329634.s008.pdf]

Supporting information

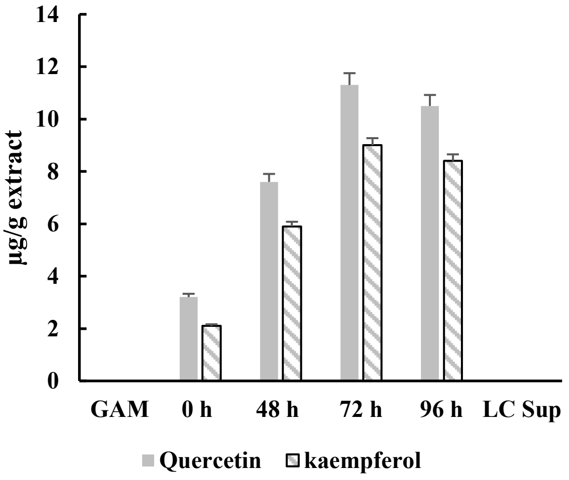

Supplementary Figure 3. Quantification of quercetin and kaempferol.

The concentrations of quercetin and kaempferol were determined in C. sativa stem extracts fermented for 0 h, 48 h, 72 h, and 96 h, as well as in controls including GAM, LC Sup, and the non-fermented 0 h sample.
