# Supplementary material for: Enhancement of antiphotoaging properties of Cannabis sativa stem water extracts by fermentation with Lacticaseibacillus casei
Source: PLoS One. 2025 Aug 14;20(8):e0329634. doi: 10.1371/journal.pone.0329634 (PMC12352839; doi:10.1371/journal.pone.0329634)
Supplement: Attachment — Submitted filename: Response_to_Reviewers.docx [file pone.0329634.s009.docx]

**Dear Reviewer #1**

We sincerely thank Reviewer 1 for the thorough and constructive review. Below, we provide detailed responses to each of the comments.

**Major Comments**

Abstract and Objectives Clarity:
The abstract concisely summarizes the study but lacks specific numerical data or comparative metrics to substantiate claims (e.g., "enhanced bioactivity"). Including key results such as specific percentage improvements in antioxidant activity or reductions in MMP-1 expression would strengthen the abstract.
The study's objectives in the Introduction section should explicitly mention the novelty of using Cannabis sativa stems, differentiating it from prior work focusing on other plant parts.

**Response:** We appreciate the reviewer’s suggestion to improve the clarity and specificity of the abstract and objectives. In response, we revised the abstract to include quantitative data, specifically stating that fermentation resulted in a 6.6% greater inhibition of MMP-1 expression and a 68.3% increase in flavonoid content compared to the non-fermented extract (line 34-35). These additions support our claim of enhanced bioactivity with precise comparative metrics. Furthermore, we added a sentence to the abstract highlighting the novelty of our study (line 31-33). In the Introduction (line 55-68), we also emphasized the underexplored nature of *C. sativa* stems by explicitly contrasting our approach with prior studies that focus on cannabinoid-rich parts of the plant. This revision clarifies the distinct contribution of our work and the rationale for investigating the stem extracts.

Experimental Design:
The choice of fermentation duration (72 hours) and conditions (e.g., shaking speed, temperature) for L. casei lacks justification. Why were these parameters chosen, and were any preliminary optimization experiments conducted?
Control comparisons: The inclusion of ascorbic acid as a positive control is appropriate, but the authors should also compare fermented and non-fermented extracts statistically to determine the significance of fermentation specifically.

**Response:** Thank you for this important comment. In response, we clarified in the Materials and Methods section that fermentation time points (0 h, 48 h, 72 h, 96 h) were selected to evaluate time-dependent changes in bioactivity, while the temperature (37 °C) and shaking speed (200 rpm) were kept constant based on standard conditions for *Lacticaseibacillus casei* growth. Although we did not conduct a full optimization study, preliminary testing indicated that fermentation for 72 hours resulted in the highest biological activity (Fig. 2). Additionally, we included statistical comparisons between fermented and non-fermented extracts across all key experiments, such as MMP-1 expression, antioxidant capacity, and ROS reduction, to determine the specific contribution of fermentation. These comparisons are reflected in the updated figures and tables with appropriate significance indicators.

Data Analysis and Representation:
The manuscript does not report effect sizes or confidence intervals for the data, which are critical for assessing biological relevance, especially in translational contexts.
Figures lack sufficient annotations to guide interpretation. For example, Figure 2 (Western blot) should indicate molecular weight markers and justify the specific signaling pathways selected.

**Response:**

We appreciate this constructive feedback. While the primary focus of our statistical analysis was on p-values, we recognize the value of reporting effect sizes and confidence intervals. Accordingly, we describe relative fold-changes within the Results section and Abstract to help interpret biological relevance. Future *in vivo* studies will incorporate confidence intervals and effect size metrics more explicitly to support translational applicability. Importantly, we have now included explicit statistical comparisons between the non-fermented extract (0 h) and the fermented samples (48 h, 72 h, 96 h) across all major experiments, including MMP-1 expression, intracellular ROS levels, antioxidant activity, and Western blot results. These comparisons are reflected in the figures (e.g., Figure 2 and Figure 3), with updated legends that clearly indicate significance levels (e.g., †p < 0.05 vs. 0 h).

Additionally, molecular weight markers (kDa) have been added to each Western blot image in Supplementary Figure 2, and the figure legend now provides justification for selecting ERK, JNK, and p65 as representative UVB-induced photoaging signaling molecules. These additions improve both the clarity and interpretability of the data.

Biological Relevance and Clinical Translation:
The in vitro model using HDFs is appropriate but limited. The discussion acknowledges this but could be improved by suggesting specific in vivo models (e.g., murine photoaging models) for future validation.
The focus solely on UVB-induced damage ignores the potential broader applicability of the extracts, such as protection against UVA or environmental pollutants.

**Response:**

We thank the reviewer for this important perspective. In response, we have expanded the Discussion section to suggest specific *in vivo* models for future validation. In particular, we propose the use of hairless mice strains such as HR-1 and SKH-1, which are well-established murine photoaging models used to study UV-induced skin damage, collagen degradation, and oxidative stress.

Additionally, we now discuss the broader applicability of the fermented *C. sativa* stem extracts beyond UVB-induced damage. We highlight their potential to mitigate skin aging caused by UVA radiation, which penetrates deeper into the dermis, and environmental stressors such as particulate matter (PM2.5) and polycyclic aromatic hydrocarbons (PAHs), both of which induce oxidative stress and inflammation. These future directions will help determine whether the antioxidant and anti-inflammatory properties observed *in vitro* can extend to complex real-world skin aging contexts.

Mechanistic Insights:
The discussion on fermentation enhancing bioactivity (e.g., flavonoid content) is convincing but speculative. Are there any specific fermentation byproducts identified that correlate with observed effects (e.g., HPLC or LC-MS data)? Without such analysis, the mechanistic claims are incomplete.

**Response:**

We appreciate the reviewer’s suggestion to strengthen the mechanistic explanation. While we did not perform untargeted metabolomic profiling (e.g., LC-MS) in this study, we fully agree that a more comprehensive analysis of fermentation-derived metabolites would provide deeper mechanistic insight. In response, we performed HPLC analysis to quantify two major flavonoid aglycones, quercetin and kaempferol, in fermented and non-fermented *C. sativa* stem extracts. The results revealed a 3.5-fold and 4.3-fold increase, respectively, after 72 hours of fermentation, which correlates well with the observed enhancement in antioxidant activity and MMP-1 inhibition. These data are now reported in the Results section and summarized in S3 Fig.

Ethical Considerations:
While the manuscript states that no ethical approval was required, the use of human-derived dermal fibroblasts demands a clearer mention of the cell line's origin and adherence to ethical standards for cell line use.

**Response:**

Thank you for raising this important point. In the revised manuscript, we have clarified that the human dermal fibroblast (HDF) cell line used in this study was obtained from the American Type Culture Collection (ATCC), a certified commercial provider. We also included a statement in the Materials and Methods section affirming that all experiments were conducted in accordance with institutional ethical guidelines and the Declaration of Helsinki. As the HDFs were commercially available and not derived from human subjects by the authors, no additional ethical approval was required.

Minor Comments

Language and Terminology:
Certain phrases, such as "fermentation significantly enhanced bioactivity," are vague. Replace with specific metrics (e.g., "fermentation increased flavonoid content by X%, enhancing antioxidant activity by Y%").
Repetition in the Introduction regarding Cannabis sativa’s underexplored potential in skincare can be condensed.

**Response:**

We appreciate the reviewer’s suggestion to improve clarity and precision. In response, we revised vague phrases such as “fermentation significantly enhanced bioactivity” throughout the Abstract and Results sections to include quantitative data. For example, we now state that fermentation increased flavonoid content by 68.3% and MMP-1 inhibition by 6.6% compared to the non-fermented extract. These changes provide a clearer representation of the observed effects. Additionally, we have revised the Introduction to to maintain the novelty of using stem extracts.

Figures and Tables:
Table 1 should include significance levels for each comparison, not just different letter labels. Use asterisks or p-values for clarity.
Figures should have high resolution in the final submission to avoid interpretive issues during review.

**Response:**

Thank you for the helpful suggestions. In response, we have updated Table 1 to include asterisk-based significance indicators in addition to the letter labels. This provides greater clarity for pairwise comparisons, particularly between the fermented and non-fermented extract groups. Furthermore, all figures have been exported at high resolution (600dpi) for final submission to ensure accurate visualization and prevent interpretive issues during review or publication.

Citations and Literature Integration:
Several references are over five years old. Consider citing recent studies on plant-based antiphotoaging agents or Cannabis sativa bioactivity.
The introduction lacks a detailed comparison with other plant extracts fermented with Lacticaseibacillus spp. Addressing this would position the study better in the context of current literature.

**Response:**

We appreciate this comment and have revised the manuscript accordingly.

To address the first point, we have updated the reference list to include recent studies published within the last five years on both plant-based antiphotoaging compounds and *Cannabis sativa* bioactivity. These include newly added citations covering the antioxidant and anti-inflammatory effects of *C. sativa* roots and seeds, as well as recent reviews on polyphenol-based anti-aging strategies.

In addition, we revised the Introduction to include a direct comparison with other plant extracts fermented using *Lacticaseibacillus spp.* Specifically, we cited recent works involving sprouted oats, yellow whey, and herbal medicines fermented with *L. casei*, all of which demonstrated enhanced bioactivity. We added a sentence highlighting that, to date, no study has evaluated *C. sativa* stems in this context, positioning our study as the first to explore this novel application.

These revisions strengthen the manuscript’s contextual grounding and clarify its contribution to the field of natural product–based skin protection research.

Formatting Issues:
Ensure uniformity in referencing styles, particularly with et al. usage.
Standardize units (e.g., μg/mL vs mg/mL) across the manuscript for consistency.
**Response:**

Thank you for your suggestion. We have carefully reviewed the manuscript to ensure consistency in referencing style and *et al.* usage in accordance with the journal’s formatting guidelines. Regarding units, we have standardized concentration values to μg/mL wherever appropriate. However, in a few cases where values would become numerically too large or small, we retained mg/mL units to preserve clarity and readability. All such instances have been checked to ensure they are scientifically appropriate and consistently formatted within their respective contexts.

Limitations Section:
While some limitations are mentioned, the manuscript should specifically discuss the absence of mechanistic validations (e.g., metabolomic analyses of fermentation products) and the lack of UVA or other stressor evaluation.

**Response:**

Thank you for pointing this out. In the revised Discussion section, we have expanded the limitations to explicitly address both points raised by the reviewer.

First, we acknowledge that untargeted metabolomic analyses such as LC-MS were not performed in this study. Although we quantified quercetin and kaempferol via HPLC, a broader profiling of fermentation-derived metabolites is essential to fully elucidate the mechanism underlying the enhanced bioactivity. We have indicated that LC-MS–based metabolomic validation is planned for future studies.

Second, we note that the current study focused exclusively on UVB-induced photoaging. As the reviewer suggested, we now discuss the importance of evaluating UVA radiation and environmental stressors (e.g., PM2.5, PAHs) in future experiments. These additions emphasize the need to validate the protective effects of fermented *C. sativa* stem extracts in more complex and physiologically relevant models.

Supplementary Information:
The supplementary data should detail the antibodies used in Western blotting, including catalog numbers and suppliers, for reproducibility.
**Response:**

We thank the reviewer for this important suggestion. In the revised manuscript, we have added Supplementary Table 1, which provides detailed information on all antibodies used for Western blotting. This information has been included to ensure full transparency and reproducibility, in line with current reporting standards.

Overall Assessment

The manuscript addresses a novel area of research with potential applications in natural skincare. However, it requires substantial revisions to improve clarity, depth, and rigor. Addressing the major comments will ensure the study's findings are robust and impactful, while minor comments focus on improving readability and presentation.

**Response:**

The manuscript addresses a novel area of research with potential applications in natural skincare. However, it requires substantial revisions to improve clarity, depth, and rigor. Addressing the major comments will ensure the study's findings are robust and impactful, while minor comments focus on improving readability and presentation.

**Dear Reviewer #2**

We sincerely thank Reviewer 2 for the thorough and constructive review. Below, we provide detailed responses to each of the comments.

Reviewer #2: The study investigates the antiphotoaging properties of fermented Cannabis sativa stem extracts using a human dermal fibroblast (HDF) model. While the authors emphasize the underexplored biological effects of cannabis stems, they also acknowledge prior research on cannabis leaves and flowers. Although stems and leaves differ significantly in their bioactive substance content, the claimed novelty of this study warrants careful consideration.
The manuscript has several strengths, including its focus on an underutilized part of the plant and its exploration of fermentation as a means of enhancing bioactivity. However, there are several critical issues and clarifying points that should be addressed before the study can be considered for publication.

**Key Comments and Questions**
1. In the section describing cytotoxicity determination, it is unclear whether all samples (including those with extracts) were UVB-irradiated. If so, please clarify the rationale behind this approach. Cytotoxicity assay should only evaluate one compound/extract at a time.
**Response:**

We thank the reviewer for this important observation. We agree that cytotoxicity should be evaluated independently of UVB-induced stress. In the revised manuscript, we have clarified that the cytotoxicity test was conducted without UVB irradiation, and all extract concentrations (0–1000 μg/mL) were tested on HDFs under normal conditions. The purpose of this assay was solely to confirm that the tested concentrations of both fermented and non-fermented extracts, as well as controls (LC sup and GAM), were non-toxic to the cells prior to UVB exposure. To address potential confusion, we have revised the description in the Materials and Methods section to clearly state that UVB irradiation was not applied during the cytotoxicity assay, and this assay was designed to select safe concentrations for subsequent UVB-based experiments.

2. On line 155, the term "CW" appears but is not defined in the methods section. If it refers to the cannabis water extract, why is it labeled "CW" here instead of "CS," as initially described?
**Response:**

Thank you for highlighting this inconsistency. In the process of revising the manuscript, we removed references to the ethanol extract and restructured the nomenclature for clarity. Accordingly, the label “CS” now refers specifically to the non-fermented (0 h) water extract used as a baseline control.

3. The LC control group, which is defined as the culture supernatant of Lacticaseibacillus casei, shows notable biological effects in several assays, including:
- MMP-1 ELISA
- ROS scavenging assays (ABTS and DPPH)
- Polyphenol and flavonoid content assays
- How do the authors interpret these results? Given that LC is a control group without plant extracts, its high activity raises concerns about the contributions of bacterial metabolites or medium components.
**Response:**

We thank the reviewer for this critical and insightful comment. Indeed, the LC control group (culture supernatant of *Lacticaseibacillus casei*) showed measurable biological activity in several assays. We interpret these results as likely attributable to bioactive microbial metabolites secreted by *L. casei* during culture, such as organic acids, bacteriocins, and fermentation-derived phenolic compounds, which are known to possess antioxidant and anti-inflammatory properties. To further explore this, we performed blank control tests using the GAM medium alone (GAM group), which showed negligible activity across assays, indicating that the observed effects in the LC group are not due to media components but rather due to metabolites produced during bacterial fermentation (Fig 2 and 3). We now highlight this interpretation in the Results and Discussion sections and emphasize that the LC group provides a baseline for evaluating the synergistic effect of *C. sativa* extract and bacterial fermentation, rather than representing a strictly inactive control.

4. While the focus on stems is unique, the inclusion of cannabis leaves and flowers in the study could have provided a comparative baseline. Were these parts excluded due to practical constraints or specific research objectives?
**Response:**

We appreciate the reviewer’s thoughtful suggestion. The exclusion of cannabis leaves and flowers was intentional and based on both scientific and practical considerations. Our primary objective was to evaluate the bioactive potential of *Cannabis sativa* stems, a part of the plant that is often discarded or underutilized. In contrast, the leaves and flowers have been extensively studied for their cannabinoid content and bioactivity in previous work.

Additionally, the inclusion of leaves and flowers in our experimental design was limited by regulatory restrictions in our region, which permit research on cannabis stems but impose additional controls on cannabinoid-rich tissues such as flowers and leaves. Therefore, we chose to focus exclusively on stems to ensure compliance while also addressing a key gap in the current literature. We have added this clarification to the revised Discussion section.

5. The manuscript mentions UVB-induced activation of ERK, JNK, and p65 as part of the photoaging model. However, it is unclear whether these signaling changes were the sole basis for evaluating photoaging. Were additional endpoints (e.g., collagen degradation or ROS production) assessed?
The activation of JNK in the UVB group appears less convincing. Could the authors provide a clearer explanation or replicate the data to confirm this pathway's role in UVB-induced photoaging?
Additionally, in the FCS group, phosphorylated p65 (p-p65) appears absent, whereas the CON group shows a baseline level of p-p65. This raises questions about the reproducibility of the data or the experimental conditions. Could the authors explain why p-p65 is entirely suppressed in FCS and not in the control?
**Response:**

We thank the reviewer for these important observations. To address the concern regarding evaluation endpoints, we conducted an additional experiment to assess intracellular ROS levels using the DCFH-DA fluorescence assay. The results confirmed that ROS levels were markedly elevated following UVB exposure and significantly reduced by fermented *C. sativa* stem extracts, particularly the 72 h sample. These data are now included in Table 1 and discussed in the revised Results and Discussion sections, demonstrating that the anti-photoaging effects of the fermented extracts extend beyond signaling protein modulation to include functional oxidative stress suppression.

To clarify the concern regarding JNK activation, we repeated the western blot analysis with freshly prepared samples. The repeated experiments showed consistent UVB-induced phosphorylation of ERK, JNK, and p65, with clear suppression by fermented extracts in a time-dependent manner. The increase in p-JNK levels, although less prominent in the initial blot, was confirmed upon repetition and validated by densitometry analysis, supporting its involvement in UVB-induced photoaging. Regarding the apparent absence of p-p65 in the FCS group (72 h), the repeated blot showed a faint but detectable signal, suggesting strong but not complete inhibition. We interpret this as a result of the high anti-inflammatory activity of the 72 h fermented extract. We have clarified in the revised manuscript that all western blot experiments were conducted in triplicate and the observed trends were reproducible.

6. The study reports a nearly two-fold increase in total flavonoid content in the fermented extract (FCS) compared to the non-fermented extract (CS). While fermentation is said to enhance flavonoid production, the specific mechanism remains unexplained. Do the authors attribute this to microbial enzymatic activity, precursor transformation, or other factors?
**Response:**

We thank the reviewer for this important question. In the revised manuscript, we now provide a mechanistic interpretation for the observed increase in flavonoid content after fermentation. We attribute the enhanced flavonoid levels primarily to microbial enzymatic activity, particularly the action of β-glucosidases and other glycoside-hydrolyzing enzymes produced by *Lacticaseibacillus casei*. These enzymes can cleave glycosidic bonds in flavonoid glycosides, converting them into more bioavailable aglycone forms such as quercetin and kaempferol.

This interpretation is supported by our HPLC analysis, which showed a significant increase in aglycone flavonoids (quercetin and kaempferol) after 72 h of fermentation (S Fig. 3). These findings suggest that the increase in total flavonoid content reflects both the enzymatic release of aglycones from glycosylated precursors and potentially increased extractability due to cell wall modification during fermentation. We have added this explanation to the revised Result section.

7. On line 322, the manuscript claims that FCS reduced UVB-induced ROS levels. However, no assay directly measuring ROS levels in the presence of UVB appears in the paper. Can the authors clarify or modify this statement?
**Response:**

We thank the reviewer for this important observation. In response, we conducted a new experiment to directly measure intracellular ROS levels following UVB exposure, using the DCFH-DA fluorescence assay. This assay was performed under the same UVB irradiation conditions used throughout the study (25 mJ/cm²). The results confirmed that UVB significantly increased ROS levels in HDFs, and that treatment with fermented *C. sativa* stem extracts, particularly the 72 h sample, markedly reduced ROS accumulation. These data have now been included in the revised Results section (Table 1 and accompanying text), and the original statement in the manuscript has been retained with this new supporting evidence.

8. - The ABTS results are described as significantly different between groups, but the data show minimal variation (e.g., 88.6% for FCS vs. 89.5% for CS). This should be addressed.
- In the DPPH assay, the LC control group demonstrated the highest scavenging potential, even outperforming the fermented extract (FCS). This observation contradicts the claim that fermentation enhances scavenging activity. How do the authors explain this?
**Response:**

We thank the reviewer for this valuable comment. In response, we repeated both the ABTS and DPPH antioxidant assays under standardized conditions using freshly prepared extracts. We also included appropriate negative controls (GAM, 0 h) and the microbial-only control (LC sup) to better interpret the effects of fermentation and microbial metabolites. The results from these new experiments are presented in the revised Table 1, and statistically analyzed using asterisk-based notation (*p < 0.05, **p < 0.01, ***p < 0.001).

In the updated ABTS results, we observed clearer and statistically significant increases in scavenging activity in the 48 h, 72 h, and 96 h fermented samples compared to the non-fermented 0 h extract, with the 72 h extract showing the most consistent enhancement. These findings are now more robust and support the interpretation that fermentation improves antioxidant potential. For the DPPH assay, although the LC sup group still exhibited notable scavenging activity, it remained significantly lower than the 72 h fermented sample, based on the new dataset. We attribute the moderate antioxidant activity in the LC group to microbial metabolites such as organic acids, as also suggested by the GAM control showing minimal activity. Importantly, LC contained no detectable flavonoids or polyphenols, confirming that the enhanced DPPH activity in fermented extracts arises primarily from fermentation-mediated increases in plant-derived antioxidant compounds. We have revised the relevant sections in the Results and Discussion accordingly.

9. -The LC group is defined as “L. casei culture without plant extracts”, yet it shows measurable levels of flavonoids and polyphenols. This finding is puzzling. Could this be due to assay interference, microbial metabolites, or contamination? Additional clarification is essential.
**Response:**

We appreciate the reviewer’s careful observation. In the revised manuscript, we address this issue by including the GAM-only control to distinguish between medium components and microbial contributions (Table 1). The GAM group showed negligible levels of flavonoids and polyphenols, indicating that the basal medium itself did not interfere with the assay.

No plant material was introduced into the LC condition at any stage, and all samples were prepared with sterile filtration. Regarding the LC Sup group, while trace levels of total polyphenols and flavonoids were detected using colorimetric methods, HPLC analysis confirmed the absence of major plant-derived flavonoids such as quercetin and kaempferol. We interpret the minor signals in the LC Sup group as likely originating from microbial metabolites that possess phenolic structures or assay reactivity with fermentation byproducts such as organic acids or amino acid derivatives. We have clarified this point in the revised draft and emphasized that the main antioxidant and antiphotoaging effects observed in fermented samples derive from plant-microbe interactions, not from L. casei alone.

Summary
The study offers an intriguing approach to enhancing the antiphotoaging properties of Cannabis sativa stems through fermentation. However, several methodological inconsistencies and data interpretation issues need to be resolved. Specifically:
- Clarification of UVB irradiation protocols.
- Addressing discrepancies in the LC group’s activity.
- Providing mechanistic insights into the effects of fermentation on flavonoid and polyphenol levels.
- Revisiting claims related to ROS assays and ABTS/DPPH results.
Once these issues are addressed, the study will provide a stronger contribution to the field of natural anti-photoaging agents.

**Response:**

We sincerely thank the reviewer for their constructive summary and detailed recommendations. In the revised manuscript, we have addressed all of the identified concerns.
